# Metabolic Adaption of Ethanol-Tolerant *Clostridium thermocellum*

**DOI:** 10.1371/journal.pone.0070631

**Published:** 2013-07-30

**Authors:** Xinshu Zhu, Jiatao Cui, Yingang Feng, Yun Fa, Jingtao Zhang, Qiu Cui

**Affiliations:** 1 Shandong Provincial Key Laboratory of Energy Genetics, CAS Key Laboratory of Biofuels, Qingdao Institute of Bioenergy and Bioprocess Technology, Chinese Academy of Sciences, Qingdao, Shandong, China; 2 Public Laboratory of Bioenergy and Biofuels, Qingdao Institute of Bioenergy and Bioprocess Technology, Chinese Academy of Sciences, Qingdao, Shandong, China; 3 University of Chinese Academy of Sciences, Beijing, China; University of Florida, United States of America

## Abstract

*Clostridium thermocellum* is a major candidate for bioethanol production via consolidated bioprocessing. However, the low ethanol tolerance of the organism dramatically impedes its usage in industry. To explore the mechanism of ethanol tolerance in this microorganism, systematic metabolomics was adopted to analyse the metabolic phenotypes of a *C. thermocellum* wild-type (WT) strain and an ethanol-tolerant strain cultivated without (ET_0_) or with (ET_3_) 3% (v/v) exogenous ethanol. Metabolomics analysis elucidated that the levels of numerous metabolites in different pathways were changed for the metabolic adaption of ethanol-tolerant *C. thermocellum*. The most interesting phenomenon was that cellodextrin was significantly more accumulated in the ethanol-tolerant strain compared with the WT strain, although cellobiose was completely consumed in both the ethanol-tolerant and wild-type strains. These results suggest that the cellodextrin synthesis was active, which might be a potential mechanism for stress resistance. Moreover, the overflow of many intermediate metabolites, which indicates the metabolic imbalance, in the ET_0_ cultivation was more significant than in the WT and ET_3_ cultivations. This indicates that the metabolic balance of the ethanol-tolerant strain was adapted better to the condition of ethanol stress. This study provides additional insight into the mechanism of ethanol tolerance and is valuable for further metabolic engineering aimed at higher bioethanol production.

## Introduction

Bioethanol produced from lignocellulosic biomass is a prospective and attractive substitute for petroleum-based liquid fuels as a sustainable and renewable source of energy [1]. Consolidated bioprocessing (CBP) is a leading option for the conversion of biomass to biofuel because this technique effectively simplifies the bioconversion process and reduces the cost of bioethanol production by integrating cellulase production, biomass saccharification, and sugar fermentation into a single step [2]. *Clostridium thermocellum*, a gram-positive thermophilic anaerobic bacterium, has been proposed as a potential candidate microorganism for bioethanol CBP production due to its high efficiency of cellulose degradation and direct production of ethanol [2], [3]. However, the industrial application of *C. thermocellum* has been hampered because of its low hemicellulose utilisation, low ethanol productivity and titre, and low ethanol tolerance [4]–[6]. Various strategies, including co-cultivation with other bacteria and metabolic engineering, have been developed to improve the ethanol production and hemicellulose utilization of *C. thermocellum* [7]–[9]. However, the low ethanol tolerance of *C. thermocellum* is still one of the major bottlenecks of its bioethanol industrialisation.

Many efforts have been made to understand the ethanol tolerance mechanism and to improve the ethanol tolerance of *C. thermocellum*. Some ethanol tolerant (ET) strains have been obtained by gradual ethanol-adaption growth, genetic engineering, and chemical or UV mutagenesis [5], [10], [11]. Using these ET strains, the mechanism of ethanol tolerance of *C. thermocellum* has been studied via analysis of membrane composition and structure [12], membrane proteomic profile [13], and genome sequence [5], [10]. Changes in the membrane proteins of *C. thermocellum* ethanol-tolerant strains, as well as fatty acid composition which have resulted in an increase of membrane rigidity, have been observed [12], [13]. A recent work indicated that increased membrane fluidity is not the sole adverse effect caused by ethanol resulting in growth inhibition, and ethanol might also denature proteins thus affecting bacterial metabolism [14]. The genome sequencing of *C. thermocellum* ET strains revealed a large number of mutation sites, and a mutation in an alcohol dehydrogenase-encoding gene appears to confer most of the ET phenotypes [5], [10]. The mutation changes the co-factor specificity of the alcohol dehydrogenase, but net ethanol oxidation does not appear to be a major detoxification mechanism [10]. Therefore, it is still not clear why a change of the co-factor specificity results in ethanol tolerance, and the contribution of other mutation sites to ethanol tolerance cannot be ruled out [10]. Some ET strains of *C. thermocellum* grow slower than the wild-type (WT) strain, and the yield of ethanol in ET strains is often lower than that in the WT strain [10], [12], [13]. These previous studies on the ET strains of *C. thermocellum* suggest that the metabolism in ET strains is significantly changed compared with the WT strain.

The “-omics” technologies, such as genomic, transcriptomic, proteomic, and metabolomic profiling, can provide deep insight into the molecular mechanisms of certain phenomena or responses [10], [13], [15], [16]. In this work, to understand the mechanism of ethanol tolerance of *C. thermocellum* more deeply, we adopted systematic metabolomics to compare the intracellular and extracellular polar-/fatty-phase metabolites in *C. thermocellum* WT and ET strains in the absence and presence of 3% (v/v) exogenous ethanol (designated as ET_0_ and ET_3_, respectively) using nuclear magnetic resonance (NMR), gas chromatography-mass spectroscopy (GC-MS), and ion chromatography (IC). Significant differences in the levels of many metabolites were observed in our analysis, which sheds new light on the mechanism of ethanol tolerance of *C. thermocellum* and provides new clues and strategies for metabolic and fermentation engineering to improve the ethanol tolerance and production of *C. thermocellum*.

## Materials and Methods

### Chemicals

Sodium chloride, K_2_HPO_4_·3H_2_O, NaH_2_PO_4_·2H_2_O and nonadecanoic acid (all analytical grade) were purchased from Guoyao Chemical Co. Ltd. (Shanghai, China) and used without further treatments. D_2_O (99.9% in D) and 4,4-dimethyl-4-silapentane-1-sulfonic acid (DSS) were purchased from Cambridge Isotope Laboratories (Miami, USA). Amino acid standards for IC analysis were purchased from AccuStandard Inc. (New Haven, Connecticut USA). Carbohydrate and organic acid standards for IC analysis were purchased from Sigma-Aldrich (St. Louis,USA). Cation standards (including K^+^, Na^+^, Mg^2+^, Ca^2+^ and NH_4_^+^) for IC analysis were purchased from the Shanghai Institute of Measurement and Testing Technology (Shanghai, China).

### Strain, Medium and Ethanol Stress Treatments

The wild-type and ethanol-tolerant strains of *C. thermocellum* ATCC 35609 were kindly provided by Prof. Jian Xu (Qingdao Institute of Bioenergy and Bioprocess Technology, Chinese Academy of Sciences). The culture media were derived from GS-2 media [17] with minor modifications (g/L, KH_2_PO_4_ 1.0, K_2_HPO_4_·3H_2_O 5.0, Urea 1.0, MgCl_2_·6H_2_O 2.5, CaCl_2_·2H_2_O 0.05, FeSO_4_·7H_2_O 0.00125, cysteine hydrochloride 3.0, cellobiose 10.0, MOPS 6.0, yeast extract 10.0, Na_3_C_6_HO_7_·2H_2_O 3, and redox indicator resazurin 0.002, pH 7.6). All media were prepared in an anaerobic cabinet with an atmosphere of mixed gases (10% CO_2_, 5% H_2_ and 85% N_2_), and the cultivations were conducted in 1000 mL anaerobic bottles with 300 mL, 400 mL and 600 mL of fresh medium for the WT, ET_0_ and ET_3_ cultivations, respectively. All cultures were grown at 60°C. The biomass was determined by optical density (OD_600_) and dry cell weight (DCW) in triplicate.

### Sample Pretreatment and Metabolite Extraction

The cultivations were stopped at the late logarithmic phase by cooling in an ice-water bath for a half hour to quench metabolic activity, and the metabolites in the samples before and after quenching were checked by NMR to ensure there was no difference (Figure S1). The cells were then harvested by centrifugation (4,000 g, 10 min, 4°C). The supernatants were frozen at −80°C as the extracellular metabolite samples for future IC analysis. The cell pellets were washed with phosphate buffer (137 mM NaCl, 2.7 mM KCl, 10 mM Na_2_HPO_4_, and 2 mM KH_2_PO_4_) 3 times.

The intracellular polar metabolites were extracted using a boiling ethanol method [18]. The precipitates were used for further metabolite extraction in fatty phase. The polar extracts were concentrated and dried by rotary evaporation and lyophilisation, and the obtained extract powder was used for further NMR analysis. The fatty phase metabolites were extracted by chloroform from the precipitates of the previous boiling ethanol extraction. Briefly, 200 mg of precipitate was added into 2.5 mL of chloroform and shaken at 160 rpm for 2 h at 37°C. The supernatant was separated by centrifugation at 9,000 g for 10 min at 4°C, and the pellet was re-extracted by adding 2.5 mL of chloroform and 1.5 mL of methanol. The supernatants from the two extractions were combined and subjected to evaporation using a rotary evaporator. Finally, 1.0 mL of chloroform was added to the dried extracts, and then the extracts were flushed with nitrogen. The extracts were kept at −80°C before GC-MS analysis.

### Metabolite Analysis

#### Intracellular polar metabolites determined by NMR

Approximately 40 mg of extracted powder of polar metabolites was added into 500 µL of 100% D_2_O together with 100 µL of phosphate buffer (100 mM, pH7.4) containing 10% D_2_O and 0.02 mM DSS. The mixture was centrifuged at 4°C (16,100 g, 5 min), and the obtained supernatant was transferred into a 5-mm NMR tube for NMR analysis. Two blanks were always prepared in parallel during extraction. All^ 1^H-NMR spectra were recorded at 298 K using a Bruker 600 NMR spectrometer (600.13 MHz for^ 1^H) equipped with a 5-mm triple resonance cryoprobe (Bruker Biospin, Germany). A standard one-dimensional noesypr1d pulse sequence was used to quantify the intracellular polar metabolites. Water suppression was achieved with a weak irradiation during the recycle delay (2 s) and the mixing time (100 ms). 64 transients were collected into 32768 data points for each spectrum with a spectral width of 12 kHz. An exponential window function with a line broadening factor of 0.5 Hz was applied to all free induction decays prior to Fourier transformation. For resonance assignment purposes, two-dimensional ^1^H-^1^H TOCSY, ^1^H-^1^H COSY, ^1^H-^13^C HSQC and ^1^H-^13^C HMBC spectra were acquired. In the COSY and TOCSY experiments, 48 transients were collected into 2048 data points for each of 256 increments, and the spectral widths were 6 kHz for both dimensions. Phase-insensitive mode was used with gradient selection for the COSY experiments, whereas the MLEV-17 was employed as the spin-lock scheme in the phase-sensitive TOCSY experiment with a mixing time of 100 ms. ^1^H-^13^C HSQC and HMBC spectra were recorded using gradient selected sequences with 200 transients and 2048 data points for each of 128 increments. The spectral widths were 6 kHz for ^1^H and 26 kHz (HSQC) or 33 kHz (HMBC) for ^13^C. The data were processed into a 4096 by 2048 matrix via Fourier transformation.

The quantification of the intracellular polar metabolites was obtained from analysis of the ^1^H-NMR spectra. These spectra were manually corrected for phase and baseline distortions using TOPSPIN (Bruker Biospin, Germany), and the spectral region (δ = 0.5–9.5) was uniformly integrated into 3166 buckets with a width of 0.003 ppm (1.8 Hz) using the AMIX package (Bruker Biospin, Germany). A region (δ = 4.67–4.90) was discarded to eliminate the effects of imperfect water presaturation. The spectral areas of each bucket were normalised to the internal reference (DSS). The absolute levels of the metabolites were calculated, as milligram per gram freeze-dried metabolite extracts, from the least overlapping NMR signals of the metabolites and the relevant DSS values with known concentrations. These semi-quantitative data were expressed in the form of the mean ± standard deviation and were also subjected to classical one-way ANOVA analysis using SPSS 13.0 software and a Turkey post-test (*p*<0.05).

#### Intracellular fatty metabolites determined by GC-MS

The total lipid in the fatty phase extracts were converted to methyl esters using 2.5 mL of 2% H_2_SO_4_-methanol (v/v) with an internal standard (nonadecanoic acid, C19:0) for quantitative determination. The fatty acid methyl esters (FAMEs) were analysed with an Agilent 7890-5975 GC-MS system (Agilent Technologies Inc., Santa Clara, USA) equipped with a 30 mm×0.25 mm×0.25 µm capillary column (Agilent HP-INNOWAS). The oven temperature was initially 100°C and increased up to 240°C over 10 min (15°C min^−1^). The split ratio was 1:20, and helium was used as the carrier gas at a flow rate of 1.0 mL min^−1^ in constant flow mode. The ion source and quadrupole temperatures were 230°C and 150°C, respectively. The mass spectrometer was operated in electron impact mode at 70 eV with a scan range of 30–400 m/z. The injection sample volume was 1.0 µL.

#### Extracellular metabolite analysed via ion chromatography

The frozen extracellular metabolite samples were thawed and filtered using a filter with a 0.22-µm pore size. Four types of extracellular targeted metabolites (i.e., amino acids, carbohydrates, organic acids and cations) were identified using an ICS-3000 system and an ICS-5000 system (Dionex Corporation, Sunnyvale, USA). The standard curves of 20 amino acids, 10 carbohydrates, 12 organic acids, and 5 cations were measured and used to quantify the corresponding extracellular metabolites. The concentrations of the metabolites in an uncultured medium were also measured as a negative control. The negative control concentrations were subtracted from the concentration of extracellular metabolites in the samples. Therefore, the extracellular metabolite contents are presented as positive or negative values, which indicate released or assimilated metabolites, respectively.

## Results and Discussion

### The Growth Patterns of Wild-type and Ethanol-tolerant Strains of *C. thermocellum* were Different

The growth and dry cell weight curves of the WT, ET_0_ and ET_3_ *C. thermocellum* cultivations are shown in Figure S2. Remarkable differences were observed among these three cultivations. Both the ET_0_ and ET_3_ cultivations displayed longer lag phases and lower maximal biomass yields than that of WT, whereas the maximal biomass of ET_3_ was only around half of that of ET_0_. These results indicate that cell growth was inhibited by ethanol, which was proven in a previous report [10]. The noteworthy divergence of macroscopic growth patterns might imply great differences of microscopic metabolic profiles.

### Differential Composition and Abundance of Intracellular Metabolites in WT, ET_0_ and ET_3_ C. *thermocellum* Cultivations were Detected

Intracellular metabolome analysis, referred to as metabolic fingerprinting, can directly provide the cellular metabolic profile and metabolic changes in specific environments [19], [20]. We analysed both the polar and fatty phase intracellular metabolites of the three phenotypes of *C. thermocellum* to investigate the intracellular ethanol-tolerant strain metabolic changes.

#### The polar metabolites

**Figure 1:**
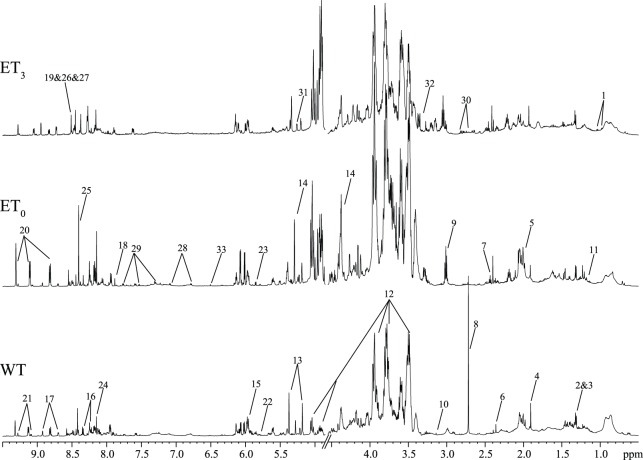
^1^H NMR spectra of the intracellular polar metabolites of *C. thermocellum*. The spectra of the wild type strain (WT) and the ethanol-tolerant strain without additional ethanol (ET_0_) and with 3% ethanol (ET_3_) were shown from bottom to top. The left halves (4.90–9.50 ppm) of all spectra are magnified two times in the Y-axis for clarification. Keys: 1, valine; 2, lactic acid; 3, threonine; 4, acetic acid; 5, glutamate; 6, pyruvate; 7, succinate; 8, dimethylamine; 9, norspermidine; 10, malonate; 11, ethanol; 12, cellodextrin; 13, phosphoenolpyruvate; 14, L-erythrose; 15, uridine monophosphate; 16, adenosine; 17, nicotinate; 18, thymidylic acid; 19, Adenosine monophosphate; 20, nicotinamide adenine dinucleotide; 21, nicotinamide adenine dinucleotide phosphate; 22, uracil; 23, uridine monophosphate; 24, inosine; 25, formate; 26, adenosine diphosphate; 27, adenosine triphosphate; 28, tyrosine; 29, tryptophan; 30, aspartate; 31, α-arabinose; 32, methanol; 33, fumarate; 34, guanine; 35, cytosine; 36, acetamide; 37, p-aminobenzoic acid; 38, uridine diphosphate glucose; 39, α-D-galactose-1-phosphate.

**Figure 2:**
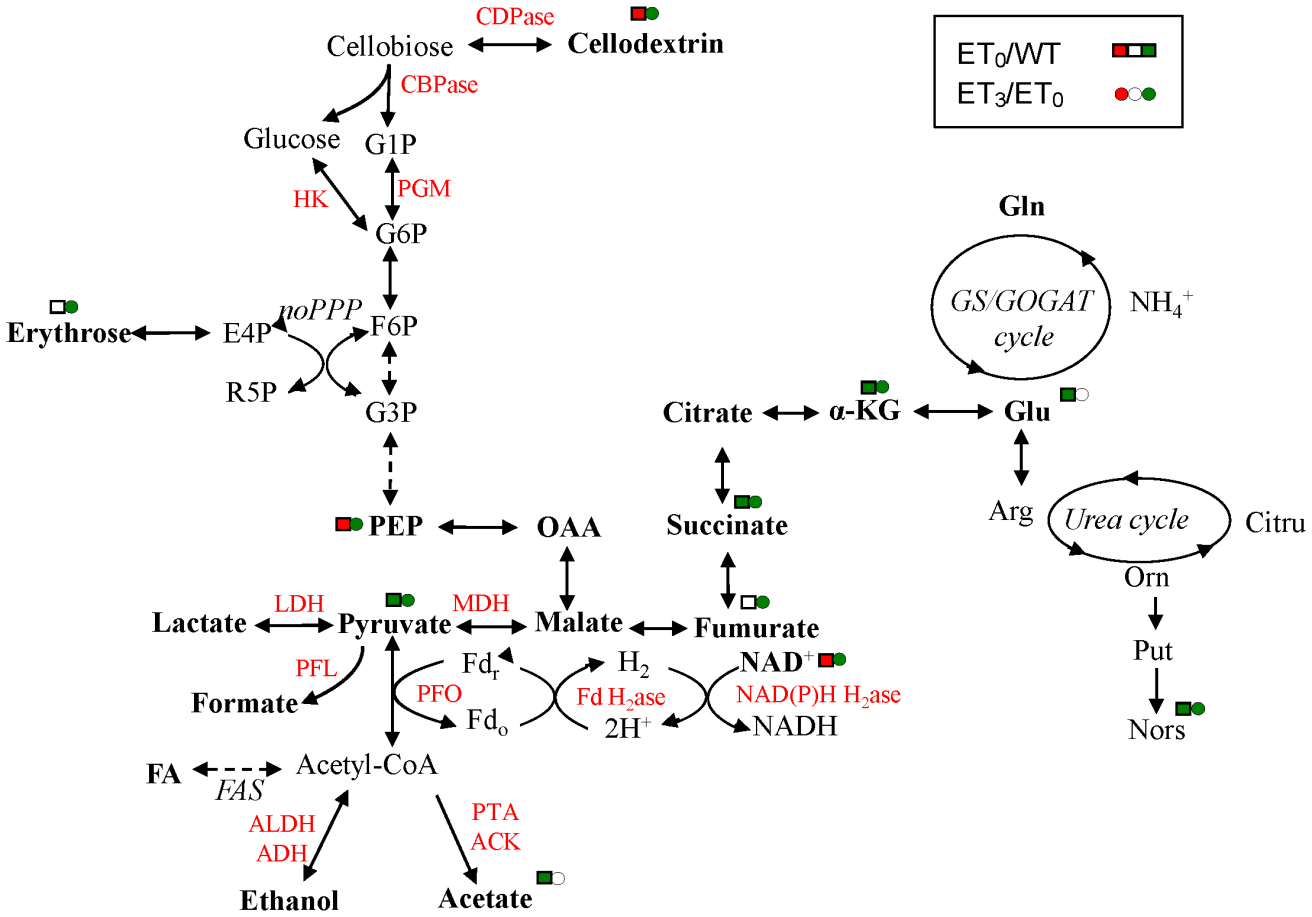
Metabolic pathway representation of intramolecular metabolite change. Significantly changed intramolecular polar metabolites in *C. thermocellum* wild-type strain (WT) and ethanol-tolerant strain without additional ethanol (ET_0_) and with 3% ethanol (ET_3_) were mapped on the existing metabolic pathways. The red and green symbols indicate significantly increased and decreased metabolites, respectively. Metabolites: G1P, glucose 1-phosphate; G6P, glucose 6-phosphate; F6P, fructose 6-phosphate; G3P, glyceraldehyde 3-phosphate; E4P, erythrose 4-phosphate; R5P, ribose 5-phosphate; PEP, phosphoenolpyruvate; OAA, oxaloacetic acid; α-KG, α-ketoglutaric acid; Glu, glutamic acid; Gln, glutamine; Arg, arginine; Citru, citrulline; Orn, ornithine; Put, putrescine; Nors, norspermidine; FA, fatty acid. Enzymes: ACK, acetate kinase; ALDH/ADH, acetaldehyde dehydrogenase/alcohol dehydrogenase; CDPase, cellodextrin phosphorylase; CBPase, cellobiose phosphorylase; HK, hexokinase; PGM, phosphoglucomutase; LDH, lactate dehydrogenase; MDH, malate dehydrogenase; PTA, phosphotransacetylase; PFL, pyruvate:formate lyase; PFO, pyruvate:ferredoxin oxidoreductase; Fd H2ase, ferredoxin hydrogenase; NAD(P)H H2ase, NAD(P)H hydrogenase.

The polar metabolites of the WT, ET_0_ and ET_3_ *C. thermocellum* cultivations were analysed by NMR (Figure 1). The metabolite resonances (Table S1) were assigned with 1D and 2D NMR data and were further confirmed with literature [21]–[23] and publicly available database data [24]. Using these data, 39 putative metabolites could be detected (Figure 1 and Table S1), of which 16 were identified and quantified (Table 1). These metabolites included amino acids, carbohydrates, organic acids/amines/alcohol, and nucleotide derivatives that are involved in various metabolic pathways (Figure 2). As shown in Table 1 and Figure 1, considerably different metabolic profiles of *C. thermocellum* were observed for the WT, ET_0_ and ET_3_. Most WT and ET_0_ metabolites displayed higher levels than those in ET_3_. This phenomenon suggested that ethanol significantly inhibited the metabolism of *C. thermocellum* even for the ET strain, which is in accordance with the observed changes in the *C. thermocellum* growth pattern (Figure S2).

**Table 1:** Quantification of the intracellular polar metabolites from the WT, ET_0_ and ET_3_ cultivations of *C. thermocellum*.

Metabolites (integral range, ppm)	Relative contents (mg/g dry-weight metabolites)		
	WT	ET_0_	ET_3_
NAD (9.30, 9.33)	1.26±0.18^a^	2.22±0.37^b^	0.25±0.14^c^
NADP (9.27, 9.30)	0.21±0.05^a^	0.18±0.04^a^	0.05±0.04^b^
nicotinate (7.57, 7.64)	0.04±0.02^a^	0.04±0.01^a^	0.01±0.01^b^
tyrosine (6.86, 6.89)	0.50±0.16^a^	0.17±0.11^b^	0.14±0.02^b^
fumarate (6.50, 6.51)	0.02±0.00^a^	0.02±0.01^a^	0.01±0.00^b^
UDPG (5.59, 5.63)	0.64±0.10^a^	0.88±0.11^b^	0.19±0.07^c^
Ery (5.28, 5.30)	0.32±0.05^a^	0.33±0.04^a^	0.11±0.04^b^
PEP (5.17, 5.19)	0.29±0.05^a^	0.35±0.03^b^	0.07±0.02^c^
cellodextrin (3.90, 3.98)	55.59±3.93^a^	108.35±15.26^b^	40.89±16.76^c^
norspermidine (2.95, 3.01)	1.61±0.12^a^	1.97±0.31^b^	0.55±0.11^c^
DMA (2.71, 2.73)	0.78±0.27^a^	0.16±0.09^b^	0.11±0.01^b^
α-KG (2.41, 2.45)	1.94±0.37^a^	1.25±0.86^b^	0.48±0.04^c^
succinate (2.38, 2.39)	0.86±0.21^a^	0.63±0.32^b^	0.19±0.03^c^
pyruvate (2.35, 2.36)	0.87±0.22^a^	0.27±0.19^b^	0.13±0.01^b^
glutamate (2.36, 2.32)	3.06±0.77^a^	1.25±0.77^b^	0.65±0.10^b^
acetate (1.89, 1.91)	2.96±1.04^a^	0.88±0.56^b^	0.42±0.05^b^

Abbreviated metabolites: NAD, nicotinamide adenine dinucleotide; NADP, nicotinamide adenine dinucleotide phosphate; UDPG, uridine diphosphate glucose; Ery, L-erythrose; PEP, phosphoenolpyruvate; DMA, dimethylamine; α-KG, α-ketoglutaric acid.

a,b,c Different letters indicated statistical significance (P<0.05) from one-way ANOVA with a Turkey post-test.

Based on extensive 2D NMR data and literature reports [23], cellodextrins were confirmed as the most abundant metabolite in the quantified metabolites of the WT, ET_0_ and ET_3_ cultivations (55.59±3.93, 108.35±15.26 and 40.89±16.76 mg/g freeze-dried weight of extracted metabolites, respectively, assuming a degree of polymerisation of 4). The accumulation of cellodextrins in *C. thermocellum* was observed in previous studies. *C. thermocellum* has been demonstrated to be able of assimilating cellodextrins hydrolysed from extracellular cellulose [25]–[27], but when cellobiose is the sole carbon source in culture media, cellodextrins are believed to be biosynthesised from cellobiose by the reverse activity of cellobiose phosphorylase and cellodextrins phosphorylase [28], [29]. However, the concentrations of cellobiose in the extracellular metabolites were quite low in both the WT or ET strains (see section of the extracellular metabolites below), which indicates that the cellodextrins accumulated in the cells cannot be explained by the reverse activity of phosphorylases when the cellobiose in the media was almost completely taken up. These results indicate that the high-concentration accumulation of cellodextrins in cells was likely an active process, and the cellodextrins potentially serve as energy storage in cells. The ET_0_ *C. thermocellum* harboured a higher concentration of cellodextrins than the WT, and the cellodextrin level in the ET_3_ bacteria was similar to that in WT, although the other metabolites in the ET_3_ bacteria were significantly decreased in comparison with the WT, which suggests the synthesis of cellodextrins in the ET strain was more active than that in the WT stain and perhaps indicates a new metabolic regulation mechanism responded to the ethanol. To check the effect of cellodextrins in *C. thermocellum*, the growths of the ET strain in absence and presence of additional cellodextrins were compared (Figure S3). Cellodextrins caused the delay of growth of ET strain, but the highest biomass was ∼ 5–10% higher than that in the absence of cellodextrins. The delay of growth was similar to the response to ethanol stress (Figure S2), whereas the higher biomass indicated the ethanol tolerance was indeed improved by cellodextrins. Further studies are needed to understand the internal mechanism of intracellular cellodextrins.

When comparing the metabolite concentrations in the ET_0_ and ET_3_ cultivations, nicotinamide adenine dinucleotide (NAD) was the most significantly changed metabolite (Table 1). The level of NAD in the ET_3_ cultivation decreased to approximately one-ninth of that in the ET_0_ cultivation, whereas the level of nicotinamide adenine dinucleotide phosphate (NADP) in the ET_3_ cultivation was approximately one-third of that in ET_0_ cultivation. These results indicate that the biosynthesis of NAD, compared to the biosynthesis of NADP, was obviously more affected by ethanol. The low level of NAD could be disadvantageous for many NAD-specific enzymes that can be very important in the process of cell growth, such as alcohol dehydrogenase, although NADP is generally more important to cell growth. Thus, this result might partially explain why the co-factor specificity change from NAD to NADP of alcohol dehydrogenase could influence the ethanol tolerance as previously reported [10]. Further studies are needed to reveal the molecular mechanism resulting in the NAD level change, which may provide a new strategy to increase the ethanol tolerance of *C. thermocellum*.

#### The fatty phase metabolites

**Figure 3:**
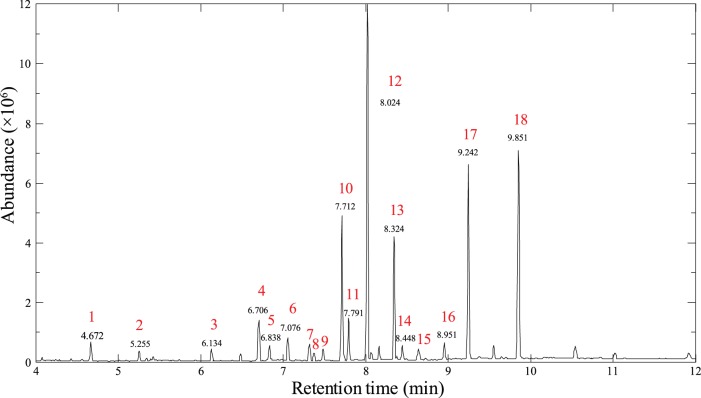
GC-MS spectrum of *C. thermocellum* for fatty phase metabolites assignment. Keys: 1, cis-13-eicosenoic acid (20:1); 2, 10-methyl-undecanoic acid (i-11:0); 3, 12-methyl-tridecanoic acid (i-13:0); 4, tetradecanoic acid (14:0); 5, plasmalogen n-14:0 (P n-14:0); 6, 13-methyl-tetradecanoic acid (i-14:0); 7, plasmalogen n-15:0 (P n-15:0); 8, pentadecanoic acid (15:0); 9, plasmalogen n-16:0 (P n-16:0); 10, 14-methyl-pentadecanoic acid (i-15:0); 11, plasmalogen i-17:0 (P i-17:0); 12, n-hexadecanoic acid (16:0); 13, 15-methyl-hexadecanoic acid (i-16:0); 14, 14-methyl-hexadecanoic acid (i-16:0); 15, heptadecanoic acid (17:0); 16, 16-methyl-heptadecanoic acid (i-17:0); 17, octadecanoic acid (18:0); 18, nonadecanoic acid (C19:0).

Fatty phase metabolites are generally the major components of the cell membrane, and thus the composition of fatty phase metabolites could provide important information about the membrane, such as the fluidity, which is considered to be a key factor in ethanol tolerance [12]. We determined the fatty acid composition of the fatty phase metabolites of C. *thermocellum* WT, ET_0_ and ET_3_ cultivations. In total, 17 metabolites were identified and quantified by GC-MS (Figure 3 and Table 2). Similar to the previous results in literature [12], the fatty phase metabolites were mainly fatty acids and plasmalogens, and the percentage of long chain fatty acids (> = 16:0) in the ET strain was higher than in the WT strain. We further detected this increasing tendency in the ET_3_ compared with ET_0_ cultivations, and the changes in fatty acid composition between the ET_3_ and ET_0_ cultivations were much less than those between the ET_0_ and WT cultivations (maximal and minimal ratios for various fatty acids were 337.24% and 22.03% for ET_0_/WT, and 139.97% and 43.79% for ET_3_/ET_0_). These results indicate that the adjustments of membrane composition under stress conditions were relatively slow compared with other metabolite changes, but some adjustments were kept in ET stains during long-term adaption. Furthermore, we observed that some fatty acids with odd carbon numbers (15:0, 17:0, P n-15:0, P i-17:0) displayed reversed changes when comparing ET_0_/WT and ET_3_/ET_0_. The percentage of these fatty acids among the total fatty acids was relatively low, so these changes might have little effect on the membrane but still reflect the responses of fatty acid biosynthesis to ethanol stress.

**Table 2:** The fatty phase metabolites of the WT, ET_0_ and ET_3_ cultivations of *C. thermocellum*.

Metabolites	Phenotype (FA/total FA%)			Percent (%)	
	WT	ET_0_	ET_3_	ET_0_/WT	ET_3_/ET_0_
*cis*-13-eicosenoic acid (20:1)	2.54±0.40	1.19±0.21	0.65±0.11	46.90*	54.17
10-methyl-undecanoic acid (i-11:0)	0.34±0.08	0.21±0.06	0.16±0.03	61.95	77.14
12-methyl-tridecanoic acid (i-13:0)	0.38±0.08	0.21±0.05	0.16±0.04	56.55	74.86
tetradecanoic acid (14:0)	4.19±0.21	1.32±0.06	0.73±0.06	31.53*	55.13*
plasmalogen n-14:0 (P n-14:0)	0.74±0.07	0.52±0.19	0.33±0.08	69.86	64.97
13-methyl-tetradecanoic acid (i-14:0)	1.18±0.24	1.12±0.32	0.64±0.12	95.54	56.81
plasmalogen n-15:0 (P n-15:0)	0.70±0.17	1.39±0.16	0.61±0.18	196.47*	43.79*
pentadecanoic acid (15:0)	0.27±0.09	0.89±0.14	0.44±0.05	337.24*	48.86*
plasmalogen n-16:0 (P n-16:0)	0.49±0.17	0.52±0.22	0.49±0.19	107.19	93.64
14-methyl-pentadecanoic acid (i-15:0)	14.78±0.36	3.26±0.39	2.48±0.52	22.03*	77.93
plasmalogen i-17:0 (P i-17:0)	5.31±0.89	1.61±0.26	1.88±0.38	29.48*	120.32
n-hexadecanoic acid (16:0)	36.39±0.89	40.26±2.56	41.18±2.70	110.64	102.28
15-methyl-hexadecanoic acid (i-16:0)	9.65±2.87	14.92±2.94	16.23±4.33	154.62	108.75
14-methyl-hexadecanoic acid (i-16:0)	2.11±0.33	2.96±0.51	2.96±0.88	140.12	100.17
heptadecanoic acid (17:0)	0.51±0.13	1.18±0.15	0.88±0.17	229.73*	74.79
16-methyl-heptadecanoic acid (i-17:0)	1.13±0.20	2.40±0.35	3.36±0.65	212.27*	139.97
octadecanoic acid (18:0)	19.30±1.84	26.08±2.84	26.76±2.56	135.12	102.64

* The significant differences are derived from a one-way ANOVA analysis (p<0.05).

### The Extracellular Metabolites of *C. thermocellum* WT, ET_0_ and ET_3_ Cultivations

The analysis of extracellular metabolites, known as metabolic footprinting, is critical for understanding microbial metabolism because extracellular metabolites are related to nutrient uptake, communication between cells and environmental factors, and metabolic end products, some of which may be particularly valuable in industrial biotechnology [20]. We identified and quantified 19 amino acids, 6 carbohydrates, 9 organic acids and 5 cations in the extracellular metabolites of the WT, ET_0_ and ET_3_ cultivations by IC (Table 3), and these metabolites represented the majority of extracellular metabolites in the media. Because we used a complex media in the cultivations, control experiments were performed to ensure that the concentrations of metabolites were not influenced by secretory enzymes (Table. S2). For most extracellular metabolites, the differences between the ET_0_ and WT cultivations were more significant than those between ET_3_ and ET_0_ with the exception of cellobiose, the major carbon source in the media (10 g/l), which was almost completely consumed in all three types of cultivations. The biomass of the ET cultivations was much less than that of the WT cultivation (Figure S2), and therefore, additional carbon sources in the ET cultivations should have been converted into extracellular metabolites. Consistently in our results, the concentrations of most extracellular metabolites in the ET cultivations were higher than those in the WT cultivation.

**Table 3:** The extracellular metabolites of the WT, ET_0_ and ET_3_ cultivations of *C. thermocellum*.

Metabolites^a^	Phenotype (ppm)			Differences	
	WT	ET_0_	ET_3_	ET_0_ vs. WT	ET_3_ vs. ET_0_
**Amino acid**					
arginine	−24.06±1.68^b^	14.15±1.48	23.80±2.72	*^c^	*
lysine	14.32±0.35	19.96±1.05	19.88±1.10	*	–d
asparagine	−0.20±0.70	1.11±0.45	1.04±0.58	*	–
glutamine	9.06±1.10	12.70±0.80	12.65±0.59	–	–
alanine	18.74±0.93	28.30±0.87	35.22±1.29	*	–
threonine	−2.53±0.20	−0.55±0.39	−0.38±0.23	*	–
glycine	8.81±0.51	7.97±0.26	7.86±0.51	–	–
valine	80.35±3.03	98.42±3.95	104.54±3.35	*	–
serine	0.93±0.03	1.69±0.11	1.84±0.11	*	–
proline	26.08±0.90	29.94±0.91	29.39±1.40	–	–
isoleucine	7.67±0.85	11.84±0.66	15.33±0.70	*	*
leucine	6.20±1.34	20.44±1.43	32.02±1.45	*	*
methionine	−0.45±0.01	−0.45±0.01	−0.21±0.01	–	*
histidine	1.59±0.12	2.52±0.17	2.24±0.17	*	–
phenylalanine	−3.57±0.14	−3.93±0.10	−3.52±0.12	–	–
glutamic acid	−33.32±1.07	−32.83±0.95	−33.08±0.79	–	–
aspartic acid	−28.29±1.50	−27.72±1.14	−25.07±1.18	–	–
cysteine	23.63±1.33	57.74±4.09	34.97±1.99	*	*
tyrosine	−2.78±0.10	7.32±0.37	5.47±0.32	*	–
**Carbohydrates**					
trehalose	−70.30±5.74	−56.97±4.02	−16.76±2.40	–	*
glucose	−360.44±25.53	−245.23±17.97	−34.39±4.05	*	*
mannose	−9.64±0.47	−5.03±0.92	−3.27±0.61	*	*
ribose	38.97±1.51	10.95±3.04	−2.51±0.31	*	*
cellobiose	−8551.70±14.74	−8548.20±17.63	−8555.90±13.14	–	–
panose	−69.97±3.73	−66.22±4.31	−43.33±6.27	–	*
**Organic acids**					
lactic acid	501.50±57.57	749.09±77.40	417.23±56.99	*	*
acetic acid	989.36±48.89	1306.56±42.95	1000.75±65.92	*	*
propionic acid	128.66±23.36	123.25±33.50	119.16±36.22	–	–
glyoxylic acid	391.48±34.44	360.62±27.8	130.51±25.61	–	*
pyruvic acid	−62.67±12.10	−62.67±19.87	−62.67±13.98	–	–
malic acid	18.52±2.66	40.97±5.83	52.08±5.15	*	–
fumaric acid	1.38±0.35	1.69±0.42	1.70±0.31	–	–
dihydroxyacetone phosphate	611.11±71.61	946.18±65.28	809.22±51.32	*	–
citric acid	−363.68±49.73	−293.94±46.65	−363.13±29.76	–	–
**Cations**					
Na^+^	−1339.22±97.41	−1097.55±75.90	−565.13±45.79	*	*
NH_4_^+^	213.36±24.10	98.20±9.08	156.08±28.57	*	*
K^+^	−427.36±74.07	−1214.52±71.79	−763.65±85.92	*	*
Mg^2+^	−164.73±12.77	−182.86±23.05	−193.36±31.88	–	–
Ca^2+^	−6.94±0.23	−9.99±0.48	−10.44±0.33	*	–

a Metabolites that were not detected among all three cultivations and control medium are not listed in the table. These metabolites included tryptophan, arabinose, galactose, lactose, malonic acid, isocitric acid, and α-ketoglutarate.

b Positive and negative values indicate amount of the released and absorbed metabolites, respectively.

c The significant differences were derived from a one-way ANOVA analysis (p<0.05).

d No significant difference was derived from a one-way ANOVA analysis (p<0.05).

It has been reported that *C. thermocellum* produces a high concentration of extracellular free amino acids [30]. In our experiments, the concentrations of 17 of 19 detected amino acids (tryptophan was not detectable in both the control medium and cultured media) in the ET cultivations were higher than those in the WT cultivation, with two exceptions, glycine and phenylalanine. Eleven secreted amino acids (the three most abundant were valine, proline and cysteine) were secreted in greater amounts by the ET strain than by the WT strain except glycine. Eight amino acids were consumed by the WT strain, 3 of which were secreted by the ET strain in contrast, whereas the other 4 amino acids, except phenylalanine, were less consumed by the ET strain. The most interesting amino acid was arginine because it exhibited the most significant change among the reversed amino acids (i.e., consumed to secreted), and the ET strain might contain mutations in the arginine biosynthesis pathway as previously reported [5]. Further studies are needed to confirm whether the mutation resulted in the overproduction and secretion of arginine in ET cultivations.

In addition to cellobiose, several carbohydrates including trehalose, glucose, mannose, and panose in media were also utilised by *C. thermocellum* (Table 3). However, unlike cellobiose, the utilisation of these sugars in the ET strain was much less than those in the WT strain. The only secreted sugar in the WT strain was ribose, but much less ribose was secreted in the ET_0_ cultivation and a small amount of ribose was taken up in the ET_3_ cultivation. Ribose is a key metabolite involved in the pentose phosphate pathway, so these changes of the extracellular ribose concentration indicated that pentose phosphate pathway was significantly affected in the ET strain.

The organic acids in metabolites include both final products and intermediates (Table 3). The detected final products included lactic acid, acetic acid, and propionic acid, and all of them were released into media. Lactic acid and acetic acid were the majority of the released final metabolites. Interestingly, the concentrations of lactic acid and acetic acid in the ET_0_ cultivation were much higher than those in both the WT and ET_3_ cultivations, unlike the released amino acids, which were released maximally in the ET_3_ cultivation. Two consumed organic acids, citric acid and pyruvic acid, were important intermediates in central metabolism. Citric acid plays key role in the TCA cycle, whereas pyruvic acid is a pivot that links glycolysis and the TCA cycle. Their uptake might save energy in the metabolism, although the initial purpose of the addition of citric acid was to prevent the precipitation of salts [17]. However, other 4 metabolic intermediates, including glyoxylic acid, malic acid, fumaric acid, and dihydroxyacetone phosphate, were significantly released into the media in all three cultivations. The phenomenon of metabolic intermediate overflow has been observed for malic acid in a previous study [31], and our results indicated that the overflow occurred for many metabolic intermediates. It has been reported that end-product-induced metabolic shifts in *C. thermocellum* [32] and the addition of ethanol changed the balance of metabolism. It is worth noticing that the metabolic overflow was more significant in the ET_0_ than ET_3_ cultivations, which suggests that the ET strain was adapted to grow with better metabolic balance in ethanol stress environments.

Five cations were detected in this study, 4 of which were taken up by *C. thermocellum*, whereas ammonium was released from cells (Table 3). The two divalent cations, Mg^2+^ and Ca^2+^, were consumed more in the ET strain than the WT strain, and the uptakes in the ET_3_ cultivation were more than those in the ET_0_ cultivation. This may be caused by the up-regulation of divalent cation transporter, as reported in a previous study [13], which is considered to be a common phenomenon in ethanol-tolerant microorganisms, and supplementation of these divalent cations may be a strategy to enhance ethanol productivity [33]. The two major monovalent cations, K^+^ and Na^+^, displayed different changes in the ET stain compared with the WT strain. In the ET strain, less Na^+^ but more K^+^ was taken up into cells. Because Na^+^ was pumped out and K^+^ was taken up into cells to maintain the membrane potential [34], [35], the ET strain should have higher membrane potential than the WT strain.

The extracellular metabolome of *C. thermocellum* provided abundant information about the nutrient uptake, metabolism, and membrane properties. Our data indicated that the major nutrient (cellobiose) was taken up completely in the ET strain but it was not converted into biomass or ethanol. Instead, greater amount of other metabolites, including amino acids and some organic acids, were produced in the ET strain, and some intermediate metabolites were more significantly overflowed. Therefore, the metabolic balance in *C. thermocellum* was not optimised for ethanol production, which might partially explain why the deletion of some by-product pathways only slightly affects the ethanol production in *C. thermocellum* [36], [37]. Careful examination and re-design of some metabolic pathways in *C. thermocellum* are needed to obtain an engineered strain with a high ethanol yield. For example, a recent successful study reported that the introduction of an exogenous pyruvate kinase increased the ethanol yield over 3-fold in *C. thermocellum* with enhanced ethanol tolerance [38]. However, the ethanol yield in the study was up to 38.8 mM ethanol, which was still far below the ethanol tolerance level of the *C. thermocellum* WT strain. Therefore, more engineering studies, including both introducing exogenous pathways and the modification of endogenous pathways, are necessary for obtaining an industrial ethanol-producing strain of *C. thermocellum*.

### Conclusion

The ethanol-tolerant strain of *C. thermocellum* displayed metabolic adaption in various metabolic pathways. The changes in the intracellular polar and fatty phase metabolites and extracellular-targeted metabolites indicated that the nutrient uptake, central metabolism, membrane structure, amino acid biosynthesis, and cation transport were adapted to ethanol stress. Cellodextrins were more significantly accumulated in the ethanol-tolerant strain as a potential anti-stress mechanism. The overflow of many intermediate metabolites indicated the imbalance of metabolic flow in *C. thermocellum,* and the ethanol-tolerant strain displayed a better adaption under the conditions of ethanol stress compared with the wild-type strain.

## Supporting Information

Figure S1 **^1^H NMR spectra of the extracted intracellular polar metabolites from wild-type** ***Clostridium thermocellum*** **before (black) and after (red) quenching experiments.**
(PDF)

Figure S2 **Growth (black) and dry cell weight curves (grey) of the WT, ET_0_ and ET_3_** ***C. thermocellum*** **cultivations.**
(PDF)

Figure S3 **The growth curves of ethanol-tolerant** ***Clostridium thermocellum*** **with and without cellodextrins.**
(PDF)

Table S1 **The NMR assignments of intracellular polar metabolites.**
(PDF)

Table S2 **The change ratio of metabolites concentrations in control experiments.**
(PDF)
