# Supplementary material for: Metabolic Adaption of Ethanol-Tolerant Clostridium thermocellum
Source: PLoS One. 2013 Jul 30;8(7):e70631. doi: 10.1371/journal.pone.0070631 (PMC3728321; doi:10.1371/journal.pone.0070631)

Figure S1. ^{1}H NMR spectra of the extracted intracellular polar metabolites from wild-type *Clostridium thermocellum* before (black) and after (red) quenching experiments.

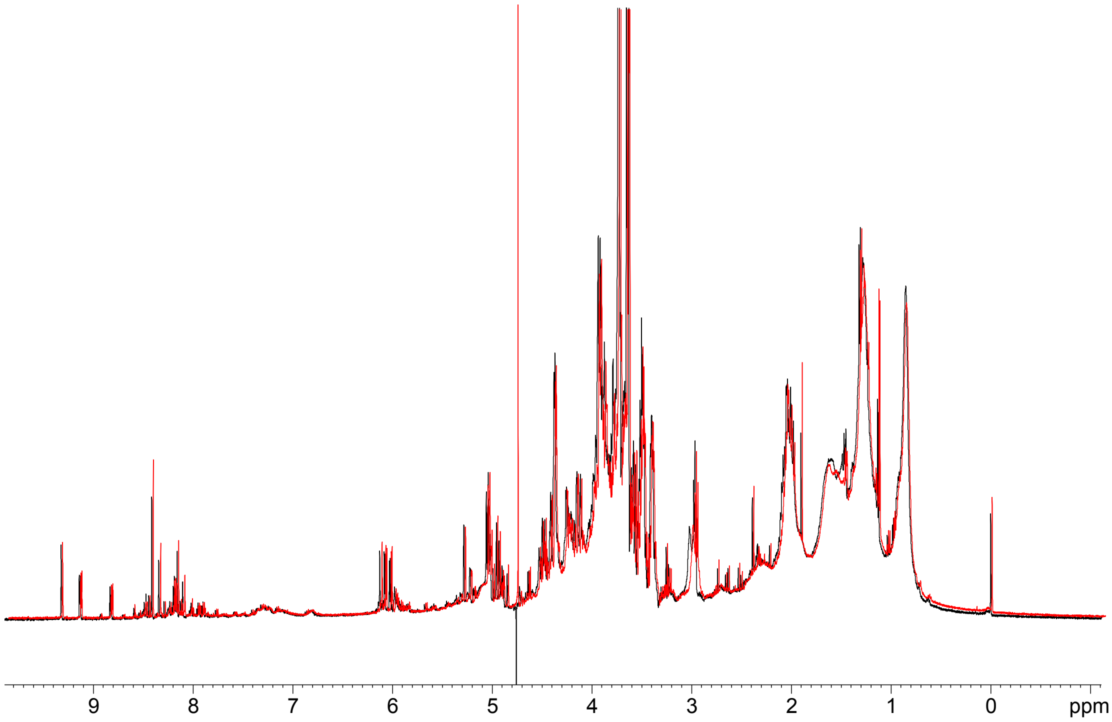

Supplement: Figure S1 — 1H NMR spectra of the extracted intracellular polar metabolites from wild-type Clostridium thermocellum before (black) and after (red) quenching experiments. (PDF) [file pone.0070631.s001.pdf]
