# Supplementary material for: Metabolic Adaption of Ethanol-Tolerant Clostridium thermocellum
Source: PLoS One. 2013 Jul 30;8(7):e70631. doi: 10.1371/journal.pone.0070631 (PMC3728321; doi:10.1371/journal.pone.0070631)

Figure S2. Growth (black) and dry cell weight curves (grey) of the WT, ET_0 and ET_3 *C. thermocellum* cultivations.

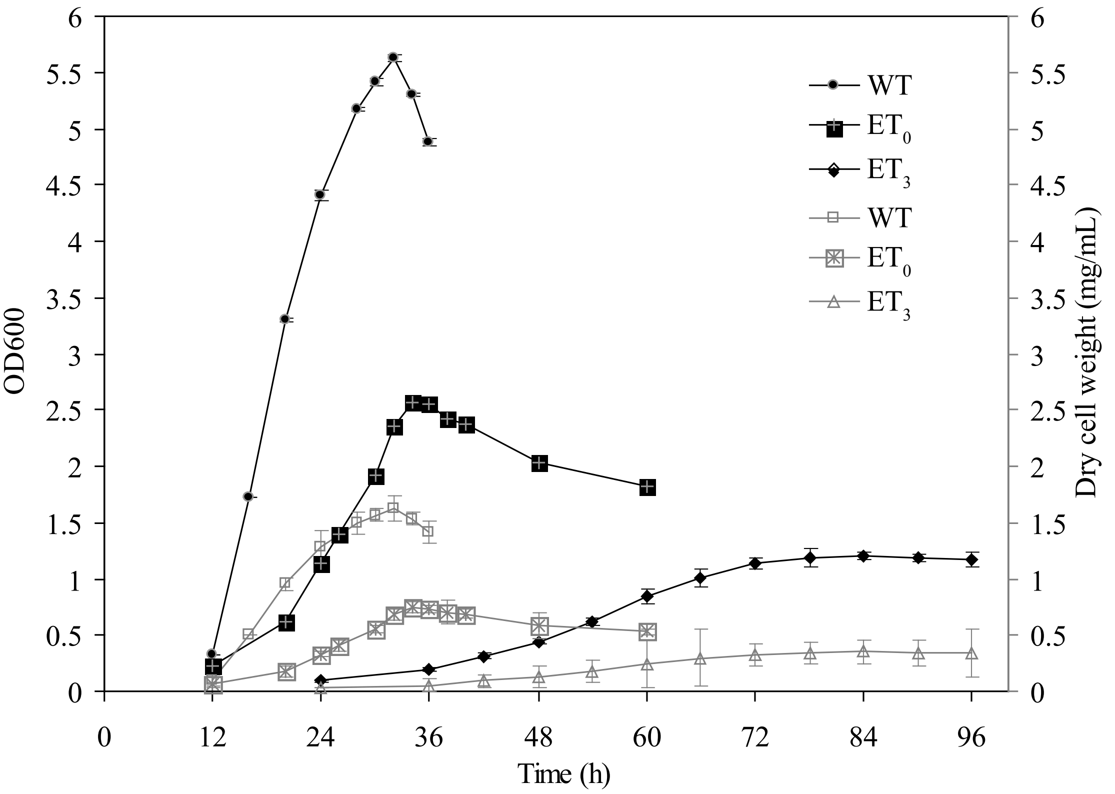

Supplement: Figure S2 — Growth (black) and dry cell weight curves (grey) of the WT, ET0 and ET3 C. thermocellum cultivations. (PDF) [file pone.0070631.s002.pdf]
