# Supplementary material for: Metabolic Adaption of Ethanol-Tolerant Clostridium thermocellum
Source: PLoS One. 2013 Jul 30;8(7):e70631. doi: 10.1371/journal.pone.0070631 (PMC3728321; doi:10.1371/journal.pone.0070631)

Figure S3. The growth curves of ethanol-tolerant *Clostridium thermocellum* with and without cellodextrins. Cellodextrins were prepared using the method in literature (Analytical Biochemistry 322 (2003) 225–232), and the degrees of polymerisation were from 2 to 9 with a mean of 4-5 which was identified by electrospray ionisation mass spectroscopy and thin layer chromatography. 5 g cellodextrins were added in the culture medium. All cultures were incubated in triplicate.

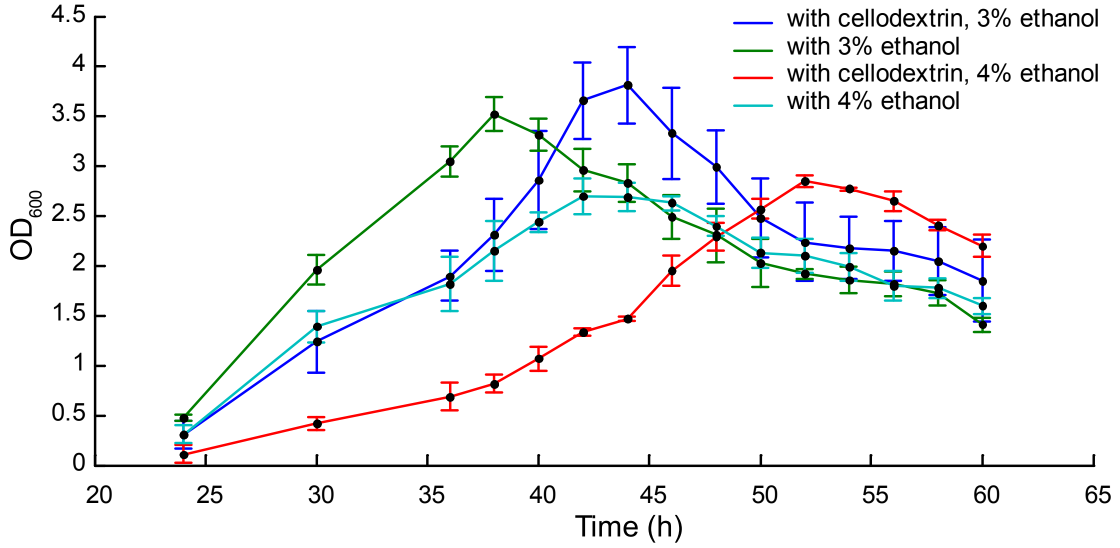

Supplement: Figure S3 — The growth curves of ethanol-tolerant Clostridium thermocellum with and without cellodextrins. (PDF) [file pone.0070631.s003.pdf]
