# Supplementary material for: Metabolic Adaption of Ethanol-Tolerant Clostridium thermocellum
Source: PLoS One. 2013 Jul 30;8(7):e70631. doi: 10.1371/journal.pone.0070631 (PMC3728321; doi:10.1371/journal.pone.0070631)
Supplement: Table S1 — The NMR assignments of intracellular polar metabolites. (PDF) [file pone.0070631.s004.pdf]

Table S1. The NMR assignments of intracellular polar metabolites.

No.	Metabolites	Group	δ^1H(multiplicity[a],Hz)	δ^{13}C
1	Valine	γ-CH_3	1.03 (d, 7.0)	22.2
		γ'-CH_3	0.98 (d, 6.8)	20.2
		β-CH_2	2.28 (m)	33.1
2	Lactic acid	3-CH_3	1.33 (d, 6.9)	24.1
		2-CH	4.12 (q)	71.2
		1-COOH		183.6
3	Threonine	4-CH_3	1.32 (d, 6.8)	24.2
		3-CH	4.18 (m)	68.4
		COOH		179.2
4	Acetic acid	2-CH_3	1.91 (s)	27.5
		1-COOH	/	181.4
5	Glutamate	2-CH	3.75 (m)	57.9
		3-CH	2.03 (m)	26.5
		4-CH	2.34 (m)	38.0
		5-COOH		175.4
6	Pyruvate	3-CH_3	2.39 (s)	38.1
		2-C=O		209.5
7	Succinate	2-CH_2	2.42 (s)	34.5
		1-COOH		174.8
8	Dimethylamine	CH_3	2.75 (s)	39.9
9	Norspermidine	3-CH_2	2.16 (m)	23.5
		2-CH_2	3.00	51.4
		1-CH_2	3.20	59.1
10	Malonate	CH	3.13 (s)	50.4
		COOH		163.0
11	Ethanol	2-CH_3	1.18 (t, 6.9)	17.1
		1-CH_2	3.66 (q)	56.8
12	Cellodextrin	1-CH	4.89, 4.92 (d, 7.8)	71.5
		2-CH	3.52, 3.59 (m)	70.8
		3-CH	3.80 (m)	78.4
		4-CH	3.59 (m)	85.0
		5-CH	3.46 (m)	79.0
		6, 6'-CH	3.80, 3.94 (m)	63.4
		α-1-CH	5.23 (d, 3.7)	95.5
		β-1-CH	4.64 (d, 7.8)	98.1
13	Phosphoenolpyruvate (PEP)	3-CH_2	5.18/5.36 (s)	102.4
		2-C		151.0
14	L-erythrose	1-CH	4.18 (m)	75.8
		2-CH	4.38 (m)	82.1
		3-CH	4.03, 3.90 (m)	69.8
		4-CH	5.29 (d, 1.5)	94.2

15	UMP	5-CH	5.96 (d,5.5)	104.4
		6-CH	7.94 (d, 8.3)	142.4
		1'-CH	5.97 (d, 3.1)	90.9
		2'-CH	4.40 (m)	76.6
		3'-CH	4.36 (m)	71.1
16	Adenosine	2-CH	8.33 (s)	*b*
		8-CH	8.25 (s)	*b*
		5'-CH	6.02 (d,6.1)	86.7
		4'-CH	4.48 (dd, 5.1, 3.4)	74.7
17	Nicotinate	2-CH	8.92 (d, 2.2)	*b*
		4-CH	8.24 (m)	*b*
		5-CH	7.58 (dd, 4.9, 7.9)	*b*
		6-CH	8.70 (dd, 1.6, 4.9)	*b*
18	d-TMP	CH	7.80 (s)	*b*
19	AMP	A2-CH	6.12 (d, 6.6)	87.2
		N8-CH	8.58 (s)	*b*
20	NAD^+	N2-CH	9.31 (s)	140.8
		N3-C		135.0
		N4-CH	8.81 (d, 8.2)	146.6
		N5-CH	8.17	129.6
		N6-CH	9.11 (d, 6.2)	143.4
		N1'-CH	6.07 (d, 5.6)	102.0
		N'2-CH	4.46 (t, 5.2)	73.1
		N'3-CH	4.37 (m)	72.8
		A2-CH	8.41 (s)	138.9
		A3-CH	8.09 (s)	*b*
		A'1-CH	6.02, 6.12(d, 6.0)	87.7
		A'2-CH	4.48 (m)	88.9
		A'3-CH	4.50 (m,)	71.0
21	$NADP^+$	N2-CH	9.25 (s)	*b*
		N6-CH	8.98 (d, 5.7)	*b*
		N4-CH	8.72 (d, 7.7)	*b*
22	Uracil	5-CH	7.52 (d, 7.6)	*b*
		6-CH	5.79 (d, 7.7)	105.1
23	UMP	12-CH	5.97 (d, 8.1)	90.7
		11-CH	7.95 (d, 8.1)	142.3
24	Inosine	2-CH	6.07 (d, 5.7)	101.8
		3-CH	4.44 (m)	89.4
		7-CH	8.18 (s)	153.2
		12-CH	8.33 (s)	143.6
25	Formate	COOH	8.44 (s)	153.7
26	ADP	A1-CH	6.13 (d, 5.7)	89.1
		A2-CH	4.51 (m)	77.2
		N2-CH	8.17 (s)	134.8

		N8-CH	8.53 (s)	141.4
27	ATP	N3-CH	8.21 (s)	157.1
		N7-CH	8.56 (s)	143.5
28	Tyrosine	3, 5CH	6.92 (d, 8.5)	*b*
		2, 6CH	7.19 (d, 8.5)	*b*
29	Tryptophan	4-CH	7.74 (d, 8.2)	*b*
		5-CH	7.15 (t, 7.4)	*b*
		6-CH	7.29 (t, 7.3)	*b*
		7-CH	7.54 (d.8.3)	*b*
30	Aspartate	β-CH_2	2.87 (dd, 7.4, 16.7)	37.1
		β'-CH_2	2.94 (dd, 4.0, 16.6)	37.1
31	α-Arabinose	1-CH	5.22 (d, 3.8)	94.1
		2-CH	3.59	65.4
32	Methanol	CH_3	3.31 (s)	50.6
33	Fumarate	2,3-CH	6.50 (s)	130.5
34	Guanine[c]	8-CH	7.65 (s)	144.3
35	Cytosine[c]	5-CH	5.79 (d, 7.7)	94.2
		6-CH	7.53 (d, 7.7)	143.1
36	Acetamide[c]	CH_3	2.05 (s)	27.1
		CO		175.4
37	p-Aminobenzoic acid[c]	3, 5CH	6.78 (d, 8.4)	117.2
		2, 6CH	7.76 (d, 8.4)	130.1
38	UDPG[c]	3-CH	5.61(dd, 3.9,)	97.4
39	α-D-galactose-1-phosphate[c]	CH	5.50 (dd, 3.3, 7.3)	96.6

[a] Multiplicity: singlet (s), doublet (d), triplet (t), quartet (q), doublet of doublets (dd), multiplet (m)

[b] The signals or the multiplicities were not determined.

[c] Tentative assignment.
