# Supplementary material for: Metabolic Adaption of Ethanol-Tolerant Clostridium thermocellum
Source: PLoS One. 2013 Jul 30;8(7):e70631. doi: 10.1371/journal.pone.0070631 (PMC3728321; doi:10.1371/journal.pone.0070631)
Supplement: Table S2 — The change ratio of metabolites concentrations in control experiments. (PDF) [file pone.0070631.s005.pdf]

Table S2. The change ratio of metabolites concentrations in control experiments. In the experiments, the culture supernatant of *Clostridium thermocellum* was passed through a filter with a 0.22 μm-pore size to remove residual cells. Then the culture was mixed with equal volume of fresh media, and then divided into two tubes. One tube was stored at -80 °C, while the other one was incubated at 60 °C for 12 h. Then the metabolites in the two tubes were compared. The results indicated that the difference of all metabolites in the two tubes was less than 3%, which was in the range of experimental errors and could be neglected in analysis.

Metabolites category	Metabolites	change ratio (%) of metabolites concentrations		
		Sample 1	Sample 2	Sample 3
amino acids	arginine	0.056	2.456	0.656
	lysine	-0.217	1.198	1.092
	asparagine	2.165	1.374	1.466
	glutamine	-0.240	0.204	0.008
	alanine	0.607	0.115	0.750
	threonine	2.257	1.978	0.858
	glycine	1.693	1.041	-0.251
	valine	0.867	1.177	1.670
	serine	-0.371	0.564	0.229
	proline	-0.393	0.373	0.239
	isoleucine	2.069	0.236	1.377
	leucine	-0.410	1.700	0.803
	methionine	-0.370	0.463	2.325
	histidine	1.055	0.538	2.394
	phenylalanine	0.099	2.053	0.916
	glutamic acid	2.118	2.190	0.163
	aspartic acid	1.250	1.015	-0.301
	cystine	-0.247	-0.378	2.156
	tyrosine	0.124	1.501	0.800
saccharides	trehalose	-0.377	0.750	1.104
	glucose	0.536	1.863	2.527
	mannose	2.452	0.247	1.511
	ribose	-0.047	1.765	2.055
	cellobiose	-0.301	0.992	0.117

	panose	0.466	0.404	1.591
organic acids	lactate	0.227	-0.172	0.923
	acetate	0.271	-0.376	2.044
	propionate	1.341	0.866	0.699
	glyoxylate	0.536	2.128	1.741
	pyruvate	2.027	2.095	0.541
	malate	0.383	1.758	0.434
	fumarate	2.464	-0.022	0.256
	dihydroxyacetone phosphate	1.698	1.768	1.162
	citrate	2.419	0.057	2.300
cations	Na^+	2.258	1.801	1.847
	NH_4^+	0.030	-0.287	-0.239
	K^+	0.417	1.471	2.085
	Mg^{2+}	1.573	1.947	0.023
	Ca^{2+}	-0.349	1.778	0.893
